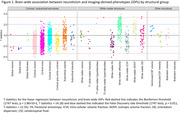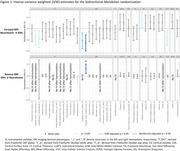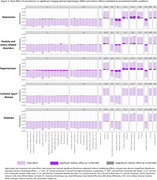# Neuroticism and brain structure: a large‐scale brain‐wide association study with mediation by health conditions

**DOI:** 10.1002/alz70856_104658

**Published:** 2026-01-07

**Authors:** Yaqing Gao, Anya Topiwala, Najaf Amin, Cornelia M Van Duijn, David J Hunter, Thomas J Littlejohns

**Affiliations:** ^1^ University of Oxford, Oxford, Oxfordshire, United Kingdom

## Abstract

**Background:**

Neuroticism, a personality trait characterized by a tendency to experience negative emotions, has been associated with cognitive decline and an increased risk of dementia. Examining its association with structural brain changes and mediators may highlight underlying biological mechanisms and potential intervention targets for at‐risk individuals.

**Method:**

We included 36,901 dementia‐free UK Biobank participants (mean [SD] age 64.4 [7.7] years). The associations between neuroticism and 1,747 structural MRI metrics were assessed using multiple linear regression, adjusted for sociodemographic, lifestyle, and imaging‐related confounders. The MRI metrics included cortical and subcortical volumes, surface area, thickness, intensities, ventricular/CSF volumes, and white matter macro‐ and microstructure. Bonferroni‐significant associations underwent bidirectional two‐sample Mendelian randomization (MR) to evaluate the evidence for a causal relationship. Causal mediation analyses were performed to assess whether a history of depression, anxiety, hypertension, ischemic heart disease (IHD), and diabetes mediated Bonferroni‐significant observational associations. These conditions were selected based on prior MR studies showing unidirectional causal effects of neuroticism on their risk, with diabetes included as a negative control due to its weak association with neuroticism.

**Result:**

Neuroticism was associated with reduced cortical volume and surface area, particularly in the frontal and limbic regions. Bonferroni‐significant associations were observed between neuroticism and lower volumes of medial frontal, subcallosal, medial orbitofrontal, and anterior cingulate cortex. Neuroticism was also associated with widespread differences in white matter microstructure, with the strongest associations observed in the thalamic radiations. For example, lower fractional anisotropy, higher diffusivity, and lower intracellular volume fraction were identified in the posterior thalamic radiations (all Bonferroni‐significant). MR analyses supported genetic associations between neuroticism and a reduced anterior cingulate cortex surface area. Hypertension mediated neuroticism's associations with cortical and white matter structures, while depression and anxiety mainly mediated white matter microstructure associations (up to 30%). Contributions from IHD and diabetes were minimal.

**Conclusion:**

Neuroticism was associated with widespread structural differences, including lower volumes in frontal and limbic regions and impaired microstructure in the thalamic radiations, partially mediated by mental and vascular conditions. Further research is needed to strengthen evidence of causality in these pathways and explore their roles in cognitive decline and dementia.